# Augmented and virtual reality usage in awake craniotomy: a systematic review

**DOI:** 10.1007/s10143-022-01929-7

**Published:** 2022-12-19

**Authors:** Mohammad Mofatteh, Mohammad Sadegh Mashayekhi, Saman Arfaie, Yimin Chen, Asfand Baig Mirza, Jawad Fares, Soham Bandyopadhyay, Edy Henich, Xuxing Liao, Mark Bernstein

**Affiliations:** 1 School of Medicine, Dentistry and Biomedical Sciences, Queen’s University Belfast, Belfast, UK; 2 Faculty of Medicine, University of British Columbia, British Columbia Vancouver, Canada; 3 Department of Neurology and Neurosurgery, McGill University, Montreal, Quebec Canada; 4 Department of Molecular and Cell Biology, University of California Berkeley, Berkeley, CA USA; 5 Department of Neurology, Foshan Sanshui District People’s Hospital, Foshan, China; 6 Department of Neurosurgery, Queen’s Hospital Romford, Romford, UK; 7 Department of Neurological Surgery, Feinberg School of Medicine, Northwestern University, Chicago, IL USA; 8 Northwestern Medicine Malnati Brain Tumor Institute, Feinberg School of Medicine, Lurie Comprehensive Cancer Center, Northwestern University, Chicago, IL USA; 9 Nuffield Department of Surgical Sciences, Oxford University Global Surgery Group, University of Oxford, Oxford, UK; 10 Clinical Neurosciences, Clinical & Experimental Sciences, Faculty of Medicine, University of Southampton, Southampton, Hampshire UK; 11 Wessex Neurological Centre, University Hospital Southampton NHS Foundation Trust, Southampton, UK; 12 Department of Medicine, McGill University, Montreal, Quebec Canada; 13 Department of Neurosurgery, Foshan Sanshui District People’s Hospital, Foshan, China; 14 Division of Neurosurgery, Department of Surgery, University of Toronto, University Health Network, Toronto, Ontario Canada; 15 Temmy Latner Center for Palliative Care, Mount Sinai Hospital, University of Toronto, Toronto, Ontario Canada

**Keywords:** Awake craniotomy, Augmented reality, Brain mapping, Mixed reality, Tumour, Virtual reality

## Abstract

Augmented and virtual reality (AR, VR) are becoming promising tools in neurosurgery. AR and VR can reduce challenges associated with conventional approaches via the simulation and mimicry of specific environments of choice for surgeons. Awake craniotomy (AC) enables the resection of lesions from eloquent brain areas while monitoring higher cortical and subcortical functions. Evidence suggests that both surgeons and patients benefit from the various applications of AR and VR in AC. This paper investigates the application of AR and VR in AC and assesses its prospective utility in neurosurgery. A systematic review of the literature was performed using PubMed, Scopus, and Web of Science databases in accordance with the PRISMA guidelines. Our search results yielded 220 articles. A total of six articles consisting of 118 patients have been included in this review. VR was used in four papers, and the other two used AR. Tumour was the most common pathology in 108 patients, followed by vascular lesions in eight patients. VR was used for intraoperative mapping of language, vision, and social cognition, while AR was incorporated in preoperative training of white matter dissection and intraoperative visualisation and navigation. Overall, patients and surgeons were satisfied with the applications of AR and VR in their cases. AR and VR can be safely incorporated during AC to supplement, augment, or even replace conventional approaches in neurosurgery. Future investigations are required to assess the feasibility of AR and VR in various phases of AC.

**Supplementary Information:**

The online version contains supplementary material available at 10.1007/s10143-022-01929-7.

## Introduction

Neurosurgery has always been at the forefront of incorporating technological advances to better surgical management, with the incorporation of augmented reality (AR) and virtual reality (VR) in recent years [5, 59, 82]. 3-D AR and VR assist with complex neurosurgical procedures in training neurosurgeons, planning surgeries, and helping with patient recovery following the surgery, respectively through projection of virtual content in the real world and generating a 3-D environment where users can fully immerse themselves [5, 82]. These two modalities have been used in multiple neurosurgical sub-specialties and have aided preoperative training and planning, surgical decision-making, intraoperative workflow, risk minimisation, postoperative clinical assessments, and rehabilitation [12, 17, 20, 30, 39–41, 45, 50, 57, 60, 63, 65, 66, 79, 89].

Computer-generated realistic images, sounds, and other sensations in VR immerse users in artificial and synthetic environments that simulate a desired situation [17, 20, 39, 40, 65]. Given that VR users have the ability to explore the artificial environment, move within it, and interact with other users [16], a simulation could be devised that enables neurosurgeons to train and plan for conducting an awake craniotomy (AC). Intraoperative brain mapping using direct electrical stimulation of cortical and subcortical regions can be performed in AC to reduce the risk of permanent loss of neurological functions in eloquent areas, such as areas associated with language, motor, or vision by real-time monitoring of neurological functions with the patient participating in different tasks [26, 29, 42, 48, 53, 54, 87, 88]. In language mapping, for example, the neurosurgeon uses direct electrical stimulation to stimulate cortical and subcortical areas, while the patient is asked in real time to name images presented to them. Perturbation or arrest of language fluency upon stimulation of certain areas marks identification of language networks and subnetworks [46]. Similarly, mapping of motor areas can be accomplished by asking patients to perform certain motor tasks [71, 76, 85]. However, other important neurological functions in social interactions, such as facial expression and eye gaze, are not mapped, mainly due to bedside restrictions in the operating room to incorporate neurophysiological functions [9]. This limitation is resolved via VR use.

Incorporating relevant information into the surgical field has been proposed for several years [1, 37]. Since its introduction to neurosurgery, in 1986, AR has become more prevalent in clinical developments such as frameless neuronavigation to help guide interventions [30, 31, 74]. Despite significant technological advances over the past few decades, current navigation approaches require manipulating surgical instruments in the surgical field while mentally reconstituting 2-D information specific to patients, such as computed tomography (CT) or magnetic resonance imaging (MRI), to 3-D anatomy in the surgical field [30]. Conventional neuronavigation tools with a screen in the operating room require exchanging surgical instruments, holding a navigation pointer in one hand, and alternating the viewing between the surgical field and the screen, therefore causing attention shift [6, 7, 28].

VR can be used in the simulation for the training of surgeons, improving understanding of complex anatomical structures, and expanding clinical assessments [39, 40, 46, 65, 82]. This is a noted advantage since it creates a risk-free setting for the clinical training of neurosurgeons across different levels to reduce the learning curve [17, 82].

A difference between the two modalities is that in an AR setup, computer-generated 2-D and 3-D images are superimposed into the vision of the real world [39, 62], whereas there is no real-world input in VR, and the user is fully immersed in a computer-generated environment [39–41]. While AR and VR have been more frequently used in other neurosurgical applications, their usage in AC remains relatively limited. In this systematic review, our primary purpose is to investigate the applications of AR and VR in AC, which can open discussions and facilitate their usage in AC. To the best of our knowledge, this paper is the first systematic review that accomplishes a synthesis of the published peer-reviewed literature on AR and VR in AC.

## Methods

### Search strategy

We conducted our systematic review based on the Preferred Reporting Items for Systematic Reviews and Meta-Analysis (PRISMA) guidelines [68] to identify published literature investigating AR and VR in AC. We conducted our electronic searches using PubMed, Scopus, and Web of Sciences from inception to May 20th, 2022, for relevant articles. The following Boolean terms were used for the search: (“augmented reality” OR “virtual reality” OR “extended reality” OR “mixed reality” OR “virtual simulation”) AND (“awake craniotomy” OR “awake brain surgery” OR “awake neurosurgery” OR “awake brain mapping” OR “awake tumour resection”) in different combinations. Details of search terms for each database are shown in Supplementary Table 1. After duplicates were removed, titles and abstracts of search results were screened, and non-related articles were excluded. Full-text articles were assessed for eligibility. Three authors (M.M., M.S.M., and S.A.) screened for relevant articles of the reference lists of selected articles to ensure no additional relevant articles were excluded.

### Inclusion and exclusion criteria

We considered articles eligible for our systematic review if they met the following conditions: (1) original articles, (2) published in English only, (3) used at least one aspect of AR and/or VR in AC, and (4) exclusively involving human subjects. The exclusion criterion was defined as (1) studies which investigated neurosurgical interventions other than AC, for example, minimally invasive procedures such as burr holes in deep brain stimulation [32, 56].

### Data extraction

Extracted data are presented in Tables 1, 2, 3, and 4 and Supplementary Table 2. All calculations were done on Microsoft Excel (version 2016; Microsoft, Redmond, WA, USA).

**Table 1:** An overview of studies’ characteristics and patients’ demographics

Study	Study type	Study period	Single/multi-centre	Patient sample size	Mean age years and SD (range)	Adults and/or paediatrics	Sex (M, F)
Mazerand et al. 2017 [61]	Prospective, case reports	September 2015–December 2015	Single-centre	10 (visual field detection), 1 (AC)	57.7 ± 21.8 (14–81) (visual field detection), 66 (AC)	Adults and paediatrics	6, 4 (visual field detection), 1 M (AC)
Bernard et al. 2018 [2]	Prospective, unblinded trial	NS	Single-centre	3	54 (NS)	Adults	2, 1
Delion et al., 2020 [18]	Prospective, open label	NS	Single-centre	30	45 (23–75)	Adults	18, 12
Casanova et al. 2021 [9]	Prospective, open label	NS	Single-centre	15	Median: 52 (25–73)	Adults	8, 7
Ille et al. 2021 [35]	Prospective	December 2019	Single-centre	5	NS (22–58)	Adults	NS
Roethe et al. 2022 [75]	Prospective	November 2017–September 2018	Single-centre	55	48.1 (15.8) (11–84)	Adults	30, 25

*AC*, awake craniotomy; *F*, female; *M*, male; *NS*, not specified; *SD*, standard deviation

**Table 2:** An overview of patients’ demographics

Study	Lesion type (*n*)	Preoperative symptoms (*n*)	Eloquent area mapping	Lesion hemisphere (*n*)	Handedness (*n*)	Lesion location (*n*)
Mazerand et al. 2017 [61]	Tumour (2), vascular (6), others (2)	Hemianopia (7), quadrantanopia (1), other (1), and generalised seizure (1, AC)	Vision, language, and motor	Left	Right (1, AC)	Parietotemporal (1)
Bernard et al. 2018 [2]	Tumour or any other surgical lesions near the language area	NS	Social cognition, language, and motor	NS	NS	NS
Delion et al. 2020 [18]	Tumour	Seizure (16), motor or speech deficits (4), cognitive deficits (2), and headache (2)	Language	Left (25) or right (5) hemispheres	Left (3) or right (27)	Frontal (15), parietal (9), temporoparietal junction (3), insula (2), and temporal lobe (1)
Casanova et al. 2021 [9]	Tumour	Seizure (9), aphasia (1), and alexia (1)	Social cognition, language, and motor	Left (11) or right (4)	Left (13) or right (2)	Frontal (8), parietal (4), temporoparietal (4). Temporoparietal junction (2), and frontotemporal insular cortex (2)
Ille et al. 2021 [35]	Tumour	NS	Language	Left (5)	NS	Frontal (2), parietal (2), insular (1) lobes
Roethe et al. 2022 [75]	Tumour (53), vascular (2)	NS	Language and motor	Left (32) and right (18)	NS	Frontal (13), parietal (10), temporal (11), insular (6), and other (15)

*AC*, awake craniotomy; *N*, number; *NS*, not specified

**Table 3:** A summary of AR/VR details from studies reviewed

Study	AR/VR	Mean surgery duration (mean AC phase)	AR/VR usage for patient and/or staff	Previous AR/VR experience (*n*)	Training provided	AR/VR use in AC phase	Comparison of AR/VR to conventional methods	AR/VR application	AR/VR setup
Mazerand et al., 2017 [61]	VR	NS	Patient	NS	NS	Intraoperative and postoperative	Yes	Visual field assessment and optic radiation mapping	Custom-made software developed by the team combined with a VR headset (Oculus DK1 and DK2, Menlo Park, CA)
Bernard et al., 2018 [2]	VR	NS	Patient and staff (neuropsychologist)	Yes — 1 patient	Yes	Intraoperative (wound closure)	No	Language and social cognition mapping	Samsung Gear VR (Samsung, Seoul, South Korea), Samsung S7 smartphone (Android), headphone, with an optional pad control and a game controller using vTime, a social network in VR enabling users to create avatars and socialising with other users in a virtual environment
Delion et al. 2020 [18]	VR	4 h 12 min (2 h 12 min)	Patient and staff (neuropsychologist)	Yes — 11 patients	Yes	Intraoperative (mapping and wound closure)	Yes	Language and social cognition mapping	Samsung Gear VR (Samsung, Seoul, South Korea) and Samsung S7 smartphone (Android) and headphone. Visual field of 96 degrees, inter-pupillary distance of 55–71 mm, latency of < 20 ms, refresh rate of 60 Hz with adjustable focus. Two applications developed: 3D motor to present 2D to 3D, and an interface to allow VR headset to communicate with a personal computer through a Bluetooth for task selection
Casanova et al. 2021 [9]	VR	4 h and 23 min (2 h 20 min)	Patient	Yes — 3 patients	Yes	Intraoperative (mapping and wound closure)	Yes	Language and social cognition mapping	High-performance VR headset, the HTC VIVE combined with eye-tracking device (Tobii Pro SDK) connected to a neuronavigational system (Brainlab)
Ille et al. 2021 [35]	AR	NS	Surgeon	NS	NS	Preoperative training	No	Training of white matter fibre dissection	A 3D model of each chosen object was added to the AR display. DTI FT of eloquent areas was used for the visualisation of the white matter
Roethe et al. 2022 [75]	AR	NS	Surgeon	NS	Yes	Intraoperative	Yes	Navigation	HUD-based AR in awake craniotomy is safe and feasible. The most advantage of it is pointer-free navigation which does not require surgeons to alternative their views from the surgical field and a display. Surgeons found AR particularly helpful in deep-seated lesions

*AC*, awake craniotomy; *AR*, augmented reality; *D*, dimension; *DTI FT*, diffusion tensor imaging fibre tracking; *HUD*, head-up display; *N*, number; *NS*, not specified; *VR*, virtual reality

**Table 4:** Major outcomes and complications from studies reviewed

Study	VR sickness experience	Follow-up (duration)	Complications and limitations with AR/VR and AC (*n*)	Patient and/or staff satisfaction	Major outcomes
Mazerand et al. 2017 [61]	NS	Yes (3 months)	NS	NS	Intraoperative visual field assessment during direct subcortical electro-stimulation using the VR headset simultaneously is a promising approach for mapping and preserving the optical radiations to prevent a permanent visual field defect during AC
Bernard et al. 2018 [2]	No	NS	Head immobilisation with a Mayfield skull clamp can limit the usage of VR headset in 360 degrees view, potentially limiting the exploration of the entire VR environment. Controlling some nonverbal communication cues, such as facial expression and eye gaze is not possible as patients are wearing the VR headset	NS	Patients undergoing AC have the ability to wear and use VR headsets and interact with an avatar pilot by a neurophysiologist. Mapping of social cognition during AC can be done using VR social networks. Future technological developments are required to address limitations
Delion et al. 2020 [18]	No	Yes (48 h)	Failure of the VR headset usage in 2 patients (1 Bluetooth failure and 1 difficulty in positioning the headset). Nine patients experienced focal intraoperative seizures, but it cannot be attributed to the VR experience. Four patients had visual discomfort (3 blurred vision and 1 lateral hemianopia)	Yes — patients reported they would repeat the procedure. Neurosurgeons, anaesthesiologists, and neuropsychologists reported no issues with using VR during the surgery	Immersing patients in a VR environment and interacting with them is possible during AC
Casanova et al. 2021 [9]	No	NS	Intraoperative focal seizure (*n* = 2) without consequences Two patients had difficulties in identifying the avatar staring at them during the training, who were excluded from this social cognition task	Yes — neurosurgeons, anaesthesiologists, neuropsychologists, and patients reported no major issues with using VR during the surgery	Immersing patients in an interactive VR environment during AC is a safe and feasible approach for mapping different cognitive functions. Simultaneous mapping of visuospatial and social functions is possible using VR
Ille et al. 2021 [35]	NA	NS	NS	Participants felt that AR/VR was a positive development with potentials for beneficial educational and clinical applications in the future	Participants rated the overall experience of AR fibre dissection, the usefulness of AR for fibre dissection course, and education in general as positive. The clinical application of AR fibre dissection was of value
Roethe et al. 2022 [75]	NA	NS	Important anatomical structures were partially or completely blocked by AR (43.6%). Technical issues were the main reason for exclusion of some cases from the AR group	66.7% found AR visualisation helpful	Integrated continuous display allowing for pointer-less navigation is the main benefit of HUD-based AR visualisation in brain tumour surgery. The highest usability is provided by Navigation view (PiP), which also blocks the operative field less frequently Surgeons operating on deep seat lesions found AR most helpful

*AC*, awake craniotomy; *AR*, augmented reality; *HUD*, head-up display; *NA*, not applicable; *NS*, not specified; *VR*, virtual reality

## Results

### Literature search results overview

**Fig. 1:**
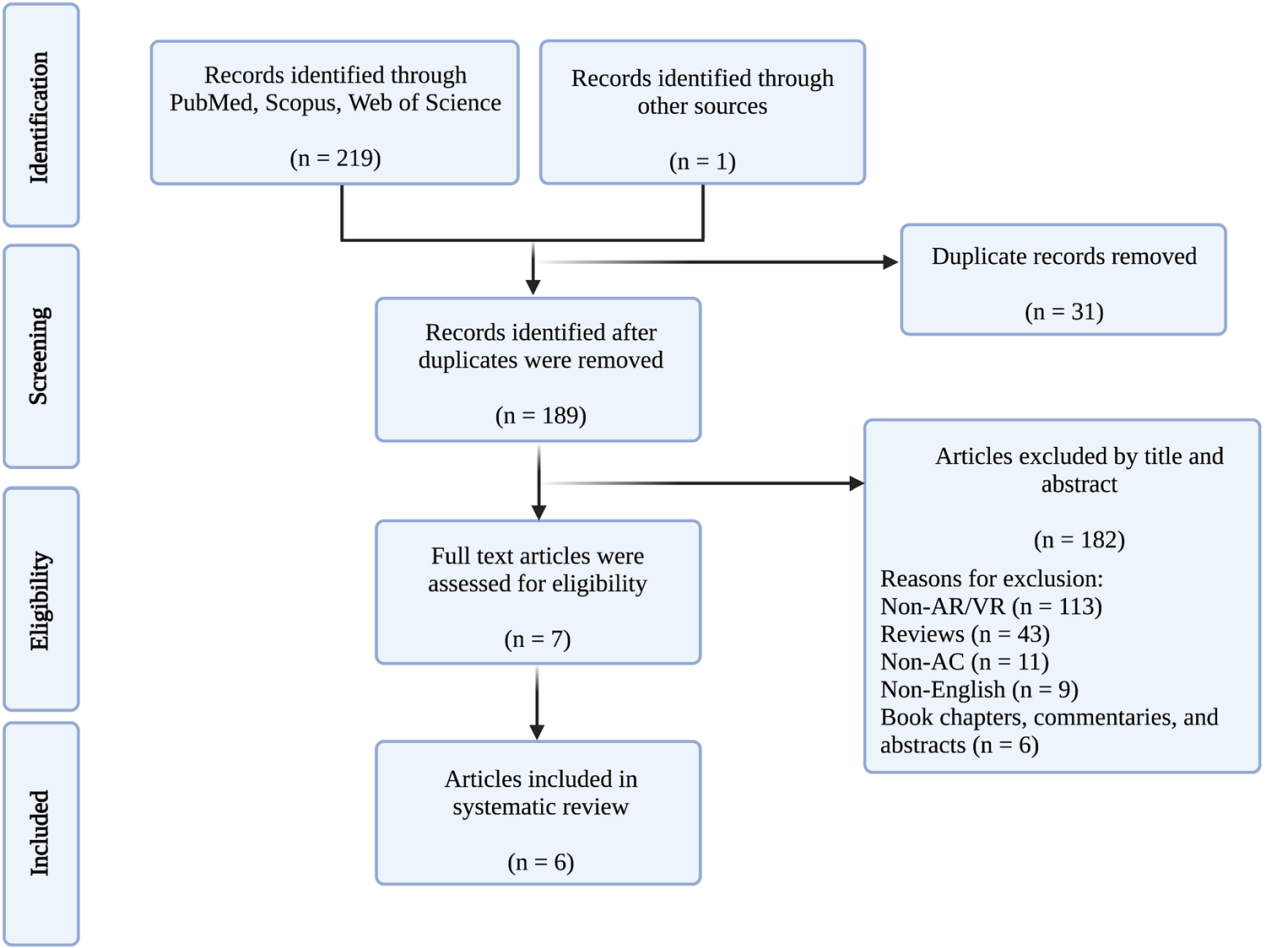
Preferred Reporting Items for Systematic Reviews and Meta-Analyses (PRISMA) flowchart demonstrating the search, screen, inclusion, and exclusion process for the current study. AC, awake craniotomy; AR, augmented reality; VR, virtual reality

Details of our search results are shown in Fig. 1. From six studies included, four papers were from France (66.67%) [2, 9, 18, 61], and two were from Germany (33.33%) [35, 75] (Supplementary Table 2).

### Study characteristics and patients’ demographics

The total sample size was 118, with the largest sample size of 55 patients reported by Roethe et al. [75], and the smallest cohort consisted of three patients [2] (Table 1). The youngest mean cohort age was noted at 45 [18], while the oldest mean age was 57.7 years [61]. The youngest patient in any cohort was 14, and the oldest was 81, both were in the study by Mazerand et al. [61]. With the exception of the 14-year-old, all studies focused on adults. Five studies specified the sex of participants [9, 18, 35, 61, 75]; there were 64 males (54.24%) and 49 females (41.53%).

The majority of pathologies operated on by AC were tumours (*n* = 108, 91.53%), followed by vascular lesions (*n* = 8, 6.78%) (Table 2). The pathology was not specified in two cases (1.69%). Five studies (83.3%) specified the lesion hemisphere and location [2, 9, 18, 61, 75]. Seventy-three lesions (61.86%) were located in the left hemisphere, and the most common lesion location was further specified as the frontal lobe (*n* = 38, 32.20%). Three studies reported preoperative symptoms in their patients, with seizure being the most common symptom recorded in 26 patients (22.03%) [9, 18, 61]. Finally, language mapping of eloquent areas was carried out in all studies. Additional mapping of motor [2, 9, 61, 75], social cognition [2, 9], and vision [61] were reported in other studies.

### Application of AR and VR in AC

Applications of AR and VR were specified with four papers studying VR [2, 9, 18, 61] and the other two investigating AR [35, 75] (Table 3). Among VR studies, two papers focused on the usage of VR for patients [9, 61], and the other two used VR for patients and staff (neuropsychologists) [2, 18]. The main uses for VR were assessing the visual field and mapping optic radiation [61] as well as mapping language and social cognitive function, including eye gazing and facial expression [2, 9, 18]. In contrast, AR was utilised for training white matter dissection [35], navigation, and visualisation of brain lesions [75]. Both AR studies involved staff, including surgeons [35, 75].

Two studies reported the mean surgery time, which were recorded as 4 h and 12 min (AC phase, 2 h and 12 min) [18], and 4 h and 23 min (AC phase, 2 h and 20 min) [9]; however, they did not specify whether AR/VR resulted in any changes in surgery duration compared to conventional methods.

Fifteen patients in three studies had previous experience using VR [2, 9, 18]. Appropriate training and instructions were provided for participants to use AR and VR in four studies [2, 9, 18, 75]. Four studies compared AR and VR to different conventional methods present [9, 18, 61, 75]. While all VR setups compared to conventional methods performed equal or better [9, 18, 61], some functions were scored equal or worse in AR compared to other conventional neuronavigation techniques [75].

### Virtual reality in awake craniotomy

While brain mapping during AC is mainly focused on language and motor areas [70, 95], VR may offer novel opportunities for mapping other complex cognitive functions, such as vision and social cognition [46]. Mazerand et al. [70] reported the first study on using VR headsets in identifying functional brain areas and functional cognitive and visual assessments during AC (Table 4). They developed a headset function similar to the Esterman test to detect homonymous and congruous visual field impairments to assess binocular visual field defects intraoperatively in AC [15]. In this setting, luminous stimuli are provided on the screen of a VR headset by selecting the points on a computer screen among those suggested by the modified Esterman test awaiting the patient’s response while direct cortical and/or subcortical stimulation was performed by the neurosurgeon in this setup. The patient communicates orally whether s/he has seen the luminous stimuli. Comparing conventional technique to VR on ten patients showed that nine patients (90%) were well-classified using the VR headset.

Bernard et al. [2] used VR to enable patients to interact with a neuropsychologist avatar for mapping their social cognition during AC. Patients observed the neuropsychologist’s avatar to comment on and reproduce gestural signs, such as *OK*, *thumbs up*, and *clap*. Bernand and colleagues concluded their study with the finding that VR could imitate complex social interactions in patients undergoing AC. The advantage of using VR to map social cognition is a fast and definite response by patients compared to other mapping approaches, such as story movies, comic strips, or interactive games, which are not compatible with a fast duration of direct electrical stimulation within a few seconds [2]. Furthermore, patient becomes an active participant in the environment rather than a mere passive observer during brain mapping procedures.

DO-80 language denomination test, where patients are asked to name 80 objects presented to them, has been extensively used for mapping language areas [11, 91], and has been incorporated into VR [9]. Delion et al. [18] duplicated the DO-80 picture naming task in the VR headset in two versions to avoid interfering with routine language mapping and AC procedures. The first version used the same images as the classic DO-80 in a virtual 2-D open space, and the other one used the 3-D version of the same objects rotating in a virtual open space. The same number of eloquent areas was identified, regardless of whether classical or VR DO-80 tests were used. However, using a VR headset successfully helped to clarify the absence of eloquent areas in three patients, which was not clear using the classical DO-80 test due to hesitation or delays in denominations. The authors speculate that VR headsets can assist patients with a better 3-D visualisation and help them focus without the distractions of the operating theatre. Furthermore, Delion and colleagues used a low-cost, high-quality, and customisable VR headset, which heated up after lengthy usage, with the phone battery acting as the rate-limiting factor [18]. While these issues did not affect their setup, such weaknesses should be considered and addressed in future investigations. In addition, placing the VR headset may interfere with the Mayfield skull clamp, which can be resolved to some extent by positioning the headset before the head holder and careful marking of incision lines.

Casanova et al. [9] used the same items as in the DO-80 language test but in a VR 3-D, similar to the setup by Delion et al. [18]. The same eloquent language areas could be identified using both conventional or VR-based language mapping, although confirmation about the absence of an eloquent area was achieved in one patient using a VR headset and not by the conventional DO-80 test. They extended their VR setup further by designing a new test for mapping eye gazing and facial expression during AC using avatars. Various types of eye movement data, such as pupil size and position as well as gaze direction, were collected using their VR headset. In their novel VR social cognition task, five different avatars were presented to patients in front of a landscape background, and each patient was asked to look at the avatar that was staring at them. Once the eye contact was established, the avatar randomly expressed a facial gesture (i.e. joy, anger, surprise) within a second after the visual contact, and patients were asked to correctly identify and describe the avatar’s emotion. This VR setup enabled establishing whether the patient had difficulties exploring the environment and locating the avatar or could not identify the facial expression after direct electrical stimulation. Eloquent areas were defined if direct electrical stimulation resulted in difficulties in exploring the VR environment, locating the avatar making eye contact, recognising the facial emotion, causing a delay, or lack of response from the patient for three times. The mean time to identify the eye contact and recognise and communicate the facial expression was 2.3 s (range, 2.0–2.5 s) and 3.2 s (range, 2.6–4.4 s), respectively, without direct electrical stimulation [18]. Particularly, the AC patient was expected to answer the questions within the time frame of each direct electrical stimulation, which lasted only 5 s.

VR sickness is accompanied by experiences such as nausea and headache [83], and may be a limiting factor for VR usage. While three studies reported no VR sickness in their participants, it is important to identify patients with risk factors to prevent adverse effects [2, 9, 18].

### Augmented reality in awake craniotomy

AR can be used for neurosurgical training. Surgical treatment of eloquent gliomas requires sufficient knowledge of the white matter to preserve these structures during tumour resection [21, 38, 80]. Current training programmes use a combination of cadaver dissection, white matter tractography on MRI, and direct electrical stimulation during AC [10, 24, 25, 33]. Ille et al. [35] used AR for evaluating fibre dissection in cadavers and in vivo tractography. By surveying 15 neurosurgeons, neurolinguistics, and neuroscientists, they demonstrated that the overall experience of using AR in fibre dissection was positive (median of 8 out of 10, mean ± standard deviation 8.5 ± 1.4, with 0 being minimum and 10 being maximum). The clinical application of AR fibre dissection was of high value, with a median of 7.0 points (7.0 ± 2.5). AR can also enable making comparisons between disease-free normal anatomy and pathological tissues during the surgery [35]. 92.3% of participants stated that using AR was reflective of their real-life clinical experience. Participants suggested incorporating more anatomical structures, inclusion of fibre tracts from both healthy subjects and patients for comparisons, and developing a more user-friendly setting such as enabling to zoom in/out and rotating the reconstitution could improve the AR setup [35].

Roethe et al. [75] investigated the clinical effectiveness of AR in brain tumour surgery by comparing 16 patients using conventional neuronavigation to 39 patients with AR-navigated microscopes after randomisation. They used MRI, diffusion tensor imaging (DTI), and brain mapping results from navigated transcranial magnetic stimulation (nTMS) in AR to insert information on the surgical field. In the AR group, surgeons used the AR function 44.4% (mean 32.2 min) of the total resection time (mean 72.5 min). In the non-AR group, pointer-based navigation resulted in frequent workflow interruptions (5–28 s each time navigation was used). Pointer utilisation frequency was significantly reduced in the AR group to 2.6 times per resection hour (0–12; SD 2.53), compared to 9.7 times per resection hour (0.8–21.6; SD 5.6) in conventional neuronavigation (*P* < 0.001). Navigation pointer was mainly used in the AR group for verifying positions during early phases of the surgery and for estimating the brain shift during advanced phases of the surgery. Head-up or head-mounted displays of AR can provide continuous integrated display, enabling pointer-less navigation. Roethe and colleagues also demonstrated that AR is most suited for deep-seated lesions (> 1 cm from the cortex), particularly during targeting small structures, defining the tumour borders, and identification of eloquent areas [75].

Neurosurgeons rated higher the spatial understanding of information (median 3.0 in AR compared to 5.0 in conventional navigation, *P* < 0.001), the visual accuracy of the overlay (median 3.0 in AR compared to median 5.0 in conventional navigation, *P* < 0.001), and visual comprehensibility (median 3.0 in AR compared to 4.0 in conventional navigation, *P* < 0.001), in conventional navigation, due to perhaps familiarity with conventional navigation compared to AR [75]. In contrast, there were no statistically significant differences in the coherence of image fusion (median 3.0 in both groups, *P* = 0.060) and relevance of visualisation (median 4.0 in AR compared to 5.0 in conventional navigation, *P* = 0.559), between the two groups. Furthermore, important anatomic structures were blocked completely or partially and were not visible in 43.6% of cases using AR. Despite such challenges, 66.7% of surgeons found AR helpful for their surgical cases, and 76.9% of those cases were categorised as deep-seated lesions [75].

According to Roethe et al. [75], neurosurgeons may have different preferences for the usage of AR during the operation. Neurosurgeons requested more AR information, especially during the initial phase of tumour resection. Also, they were more comfortable with using AR visualisation as guidance for the entire tumour removal. Therefore, personal preferences can affect the usage of AR and VR, and such variations should be considered to promote the usage of such technologies.

## Discussion

**Fig. 2:**
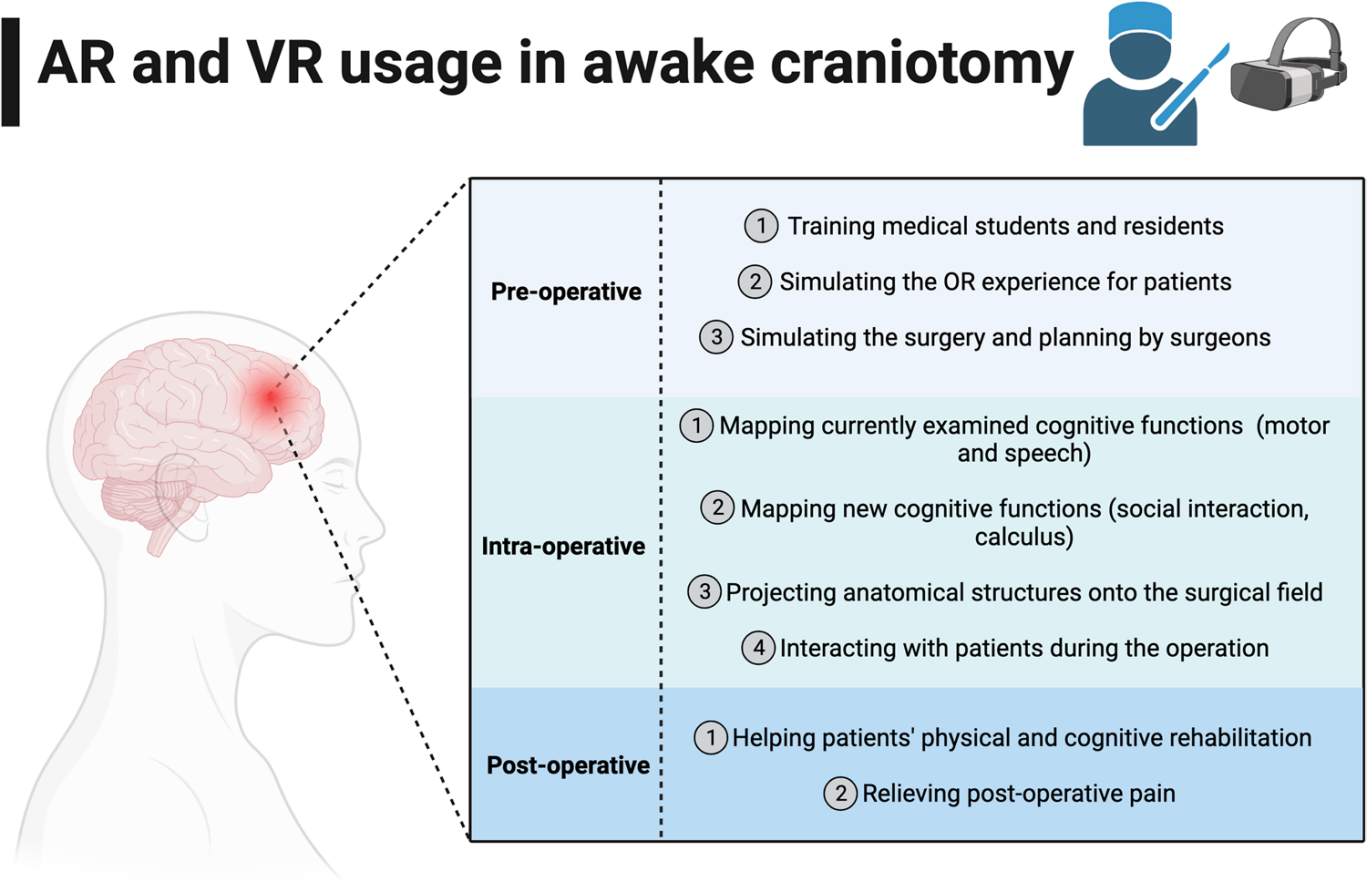
A schematic summary of AR and VR usage at preoperative, intraoperative, and postoperative phases of AC. AR, augmented reality; OR, operating room; VR, virtual reality

AR and VR are innovative domains of medicine with growing applications in neurosurgery, including training, patient care, operative planning, intraoperative assessments, and postoperative care [8, 19, 36, 37, 52, 55, 58, 69, 86, 90]. AR and VR can be used during the preoperative phase of AC (i.e. for example, in training residents and medical students, and simulating the operating room experience for patients), in the intraoperative phase (i.e. mapping cognitive functions), and in the postoperative phase (i.e. patient rehabilitation) (Fig. 2).

While language and motor mapping have been routinely performed during AC, non-verbal and non-motor mappings — such as mapping of the visual field and social cognition — have been limited due to challenges with adapting classical bedside tests to the restrictive environment of the operating room during AC [2, 9, 92]. Such associated challenges make the prediction of postoperative optic visual field defects difficult [84]. Multiple approaches, such as tractography-based neuronavigation, visual evoked potentials, and direct subcortical stimulation, have been suggested for mapping optic radiations [23, 44]. Furthermore, impairments of other non-verbal and non-motor cognitive processes, such as social cognition, including body language, empathy, facial expression, and theory of mind after neurosurgery, can be a source of concern [78]. Patients with postoperative social cognitive impairments can have difficulties in conceptualising and understanding thoughts, beliefs, desires, behaviours, and intentions of other people, and such defects have been mistakenly attributed to impairments in language and executive functions with few studies addressing them [34, 64, 72, 77]. Various tests can be designed and implemented using VR to assess domains other than language and motor during AC.

### Current state of using AR and VR in neurosurgery

VR generates realistic images and sounds to simulate users’ physical presence in a virtual environment to develop new tests for evaluating neurological functions in AC, for example by using avatars mimicking humans [9]. Using avatars to interact and socialise in VR has been reported previously in cognitive neuroscience [27]. Participants perceived avatars similar to humans and connected with them as they did with humans in the real world because social norms in the VR environment, including gender, personal space, and eye gaze, are governed by similar rules to the physical world; therefore, making their usage for non-verbal cognitive processes, such as empathy and theory of mind possible [4, 94]. Multiple other tasks testing other domains, such as auditory association, calculus, working memory, praxis, and gnosias, can be assessed using VR [46]. For example, patients can be asked to hear an animal’s sound and name an animal to assess the auditory association and perform arithmetic tasks to assess calculus, working memory, and attention [46]. We suggest that the development of future interactive VR environments can assess different domains within the same task to facilitate the preservation of multiple eloquent areas. Furthermore, VR enables investigating multiple variables such as age, race, sex, and expression, which cannot be tested in a physical world during AC.

Multiple AR-based approaches, such as image projection, head-up display (i.e. transparent displays that present data without requiring users to look away from their viewpoint), head-mounted display (i.e. devices which are worn on the head or as part of a helmet), monitor- and tablet-based visualisation, and image insertion into the surgical field, have been investigated [3, 13, 14, 19, 30, 39, 47, 49, 62, 73, 81, 93].

AR in neurosurgery involves an overlay of the necessary information, such as CT/MRI and functional information, augmented onto the surgical field [63]. Thereby, AR can provide crucial information such as tumour boundaries and risky adjacent structures to reduce surgical risks, reduce intraoperative cognitive load, and provide information for the entire surgical team [19, 37, 47, 75]. Multiple factors have been reported to influence the suitability of cases for AR and VR. Previous investigations have shown that projection techniques are best suited for superficial brain tumours [37], whereas head-up AR techniques are suitable for small deep-seated lesions [45], despite a reduction in depth accuracy [60].

### Challenges and future direction of AR and VR in neurosurgery

There are some challenges associated with AR and VR use in neurosurgery, including registration errors, depth perception errors, temporal asynchrony in visual and tactile modalities, and blocking of visual fields [30].

The quality of AR visualisation is significantly dependent on the quality of the CT and MRI data as well as successful image fusion [43, 75]. Resolution of the inserted image and segmentation is particularly challenging in neurosurgery as small diameter vessels and nerves are poorly delineated in original data [75]. Therefore, challenges associated with demarcating anatomical structures are not solved using AR. From the neurosurgeons’ perspective, pointer-free navigation during tissue preparation and tumour resection is the most suitable feature associated with AR and VR [75]. Using AR and VR can eliminate having alternative views of the surgical field to combine physical and virtual information. One of the challenges in accuracy of neuronavigation is the brain shift during AC which is present in both conventional and AR-based navigations. This can be addressed by using intraoperative imaging and updating the feeding data to AR [51].

Fade-in display of surgical information and pointer-free navigation may pose some challenges, such as completely or partially blocking the view of the surgical field and impairing depth assessment. One study demonstrated that most otolaryngology expert participants preferred the display of information in the periphery over AR visualisation in the focus level due to the distraction effect and visual occlusion [22]. Other restrictions associated with AR and VR technology include an overlay of working and viewing fields, the lack of 3-D depth in the AR, and visualisation offsets caused by MRI data resolution. One of the common problems associated with AR was 2-D versus 3-D visualisation of surgically relevant information during the surgery [75]. AR utilisation can be disruptive and challenging, especially for inexperienced users, as surgeons require training to read and apply relevant information [75]. To overcome these challenges, future studies can focus on designing user-friendly interfaces to minimise the mental and visual distractions for the surgeons. Despite these challenges, 3-D displays of various datasets over the surgical field and advances in robotic surgery, registration, and display hold promise for the use of AR and VR in the operating room in neurosurgery.

Furthermore, mapping more complex cognitive functions, such as emotions in AC, ideally requires preoperative screening to establish the baseline characteristics of the patient as well as postoperative monitoring and rehabilitation [46]. It has been shown that AC patients experience less stress, anxiety, and depression in different phases of the operation [67]. We suggest that VR can be used to assess the intraoperative level of anxiety and stress and reduce them, for example by having the patient interacting with virtual avatars.

## Limitations

This systematic review is subject to some limitations. AR and VR in AC are relatively novel concepts reflected in the limited number of publications. Our review was limited to papers published in English, and there was heterogeneity in the articles reviewed. The patient numbers were low, and the number of participants in each study and data collection period varied widely. AR and VR technologies are not widely used in all neurosurgical settings, especially in countries with resource restraints. This was reflected in the fact that all studies included in this review were from high-income countries. Furthermore, participants in included studies were patients and surgeons, and the studies reviewed had different inclusion and exclusion criteria. No study investigated both AR and VR in the same group of participants. Studies focused on different phases of AC, namely preoperative and intraoperative, and no study was done at the postoperative phase of AC. Therefore, future studies are required to evaluate AR and VR at all phases of AC. Despite such limitations, the current review can be a useful addition to understanding AR and VR in neurosurgical procedures and prompt further research.

## Conclusion

The technologies concerning AR and VR are rapidly advancing in neurosurgery, and they can be used at different phases of AC by patients and surgeons. As the technological capability of AR and VR advances, neurosurgeons are finding more applications in training and patient interaction, within and out of the operating theatre. AR and VR have been tested against existing protocols and have been shown to generate equal or better outcomes in some studies. While the benefits of AR and VR certainly outweigh their risks, multiple factors, such as case selection, technology availability, visualisation, and technical limitations, should be considered when using AR and VR in the neurosurgical operating theatre. Results from studies included in this review can only be indicative of certain domains in AR and VR, and further technical improvements are required to eliminate existing problems with AR and VR. Development of future AR and VR environments can be customised for patients and surgeons to assess specific networks and domains that they desire to be preserved. In addition, more training is required to ensure competencies are achieved among neurosurgeons and trainees. Future prospective multi-centre studies with long-term follow-up and larger sample sizes are required to assess patient outcomes in AR and VR compared to conventional approaches.

### Supplementary Information

Below is the link to the electronic supplementary material.

Supplementary file1 (DOCX 30 KB)

Supplementary file2 (DOCX 15 KB)

Supplementary file3 (DOCX 17 KB)

## Abbreviations and acronyms

AC: Awake craniotomy
AR: Augmented reality
CT: Computed tomography
DTI: Diffusion tensor imaging
HMD: Head mounted display
HUD: Head up display
MRI: Magnetic resonance imaging
NA: Not applicable
NS: Not specified
nTMS: Navigated transcranial magnetic stimulation
PRISMA: Preferred Reporting Items for Systematic Reviews and Meta-Analysis
VR: Virtual reality

## References

[1] Asano K, Katayama K, Kakuta K, Oyama K, Ohkuma H (2017). Assessment of the accuracy and errors of head-up display by an optical neuronavigation system in brain tumor surgery. Oper Neurosurg (Hagerstown).

[2] Bernard F, Lemée JM, Aubin G, Ter Minassian A, Menei P (2018). Using a virtual reality social network during awake craniotomy to map social cognition: prospective trial. J Med Internet Res.

[3] Besharati Tabrizi L, Mahvash M (2015). Augmented reality-guided neurosurgery: accuracy and intraoperative application of an image projection technique. J Neurosurg.

[4] de Borst AW, de Gelder B (2015). Is it the real deal? Perception of virtual characters versus humans: an affective cognitive neuroscience perspective. Front Psychol.

[5] Cannizzaro D, Zaed I, Safa A, Jelmoni AJM, Composto A, Bisoglio A, Schmeizer K, Becker AC, Pizzi A, Cardia A, Servadei F (2022) Augmented reality in neurosurgery, state of art and future projections. A systematic review. Front Surg 9. 10.3389/fsurg.2022.864792 10.3389/fsurg.2022.864792 PMC8961734 35360432

[6] Carl B, Bopp M, Benescu A, Saß B, Nimsky C (2020). Indocyanine green angiography visualized by augmented reality in aneurysm surgery. World Neurosurgery.

[7] Carl B, Bopp M, Saß B, Pojskic M, Voellger B, Nimsky C (2020). Spine surgery supported by augmented reality. Global Spine J.

[8] Carl B, Bopp M, Voellger B, Saß B, Nimsky C (2019). Augmented reality in transsphenoidal surgery. World Neurosurg.

[9] Casanova M, Clavreul A, Soulard G, Delion M, Aubin G, Ter Minassian A, Seguier R, Menei P (2021). Immersive virtual reality and ocular tracking for brain mapping during awake surgery: prospective evaluation study. J Med Internet Res.

[10] Catani M, Thiebaut de Schotten M (2008). A diffusion tensor imaging tractography atlas for virtual in vivo dissections. Cortex.

[11] Chang W-H, Pei Y-C, Wei K-C, Chao Y-P, Chen M-H, Yeh H-A, Jaw F-S, Chen P-Y (2018). Intraoperative linguistic performance during awake brain surgery predicts postoperative linguistic deficits. J Neurooncol.

[12] Chidambaram S, Stifano V, Demetres M, Teyssandier M, Palumbo MC, Redaelli A, Olivi A, Apuzzo MLJ, Pannullo SC (2021). Applications of augmented reality in the neurosurgical operating room: a systematic review of the literature. J Clin Neurosci.

[13] Cho J, Rahimpour S, Cutler A, Goodwin CR, Lad SP, Codd P (2020). Enhancing reality: a systematic review of augmented reality in neuronavigation and education. World Neurosurg.

[14] Contreras López WO, Navarro PA, Crispin S (2019). Intraoperative clinical application of augmented reality in neurosurgery: a systematic review. Clin Neurol Neurosurg.

[15] Crabb DP, Fitzke FW, Hitchings RA, Viswanathan AC (2004). A practical approach to measuring the visual field component of fitness to drive. Br J Ophthalmol.

[16] Dadario NB, Quinoa T, Khatri D, Boockvar J, Langer D, D’Amico RS (2021). Examining the benefits of extended reality in neurosurgery: a systematic review. J Clin Neurosci.

[17] Davids J, Manivannan S, Darzi A, Giannarou S, Ashrafian H, Marcus HJ (2021). Simulation for skills training in neurosurgery: a systematic review, meta-analysis, and analysis of progressive scholarly acceptance. Neurosurg Rev.

[18] Delion M, Klinger E, Bernard F, Aubin G, Minassian AT, Menei P (2020). Immersing patients in a virtual reality environment for brain mapping during awake surgery: safety study. World Neurosurg.

[19] Deng W, Li F, Wang M, Song Z (2014). Easy-to-use augmented reality neuronavigation using a wireless tablet PC. Stereotact Funct Neurosurg.

[20] Denmark T, Fish J, Jansari A, Tailor J, Ashkan K, Morris R (2019). Using virtual reality to investigate multitasking ability in individuals with frontal lobe lesions. Neuropsychol Rehabil.

[21] De Witt Hamer PC, Hendriks EJ, Mandonnet E, Barkhof F, Zwinderman AH, Duffau H (2013). Resection probability maps for quality assessment of glioma surgery without brain location bias. PLoS ONE.

[22] Dixon BJ, Daly MJ, Chan HH, Vescan A, Witterick IJ, Irish JC (2014). Inattentional blindness increased with augmented reality surgical navigation. Am J Rhinol Allergy.

[23] Duffau H, Capelle L, Denvil D, Sichez N, Gatignol P, Taillandier L, Lopes M, Mitchell M-C, Roche S, Muller J-C, Bitar A, Sichez J-P, van Effenterre R (2003). Usefulness of intraoperative electrical subcortical mapping during surgery for low-grade gliomas located within eloquent brain regions: functional results in a consecutive series of 103 patients. J Neurosurg.

[24] Duffau H, Gatignol P, Mandonnet E, Peruzzi P, Tzourio-Mazoyer N, Capelle L (2005). New insights into the anatomo-functional connectivity of the semantic system: a study using cortico-subcortical electrostimulations. Brain.

[25] Duffau H, Moritz-Gasser S, Mandonnet E (2014). A re-examination of neural basis of language processing: proposal of a dynamic hodotopical model from data provided by brain stimulation mapping during picture naming. Brain Lang.

[26] Dziedzic T, Bernstein M (2014). Awake craniotomy for brain tumor: indications, technique and benefits. Expert Rev Neurother.

[27] Georgescu AL, Kuzmanovic B, Roth D, Bente G, Vogeley K (2014). The use of virtual characters to assess and train non-verbal communication in high-functioning autism. Front Hum Neurosci.

[28] Gerard IJ, Kersten-Oertel M, Hall JA, Sirhan D, Collins DL (2020). Brain shift in neuronavigation of brain tumors: an updated review of intra-operative ultrasound applications. Front Oncol.

[29] Ghimire P, Lavrador JP, Baig Mirza A, Pereira N, Keeble H, Borri M, Furlanetti L, Brogna C, Jarosz J, Gullan R, Vergani F, Bhangoo R, Ashkan K (2021). Intraoperative mapping of pre-central motor cortex and subcortex: a proposal for supplemental cortical and novel subcortical maps to Penfield’s motor homunculus. Brain Struct Funct.

[30] Guha D, Alotaibi NM, Nguyen N, Gupta S, McFaul C, Yang VXD (2017). Augmented reality in neurosurgery: a review of current concepts and emerging applications. Canadian Journal of Neurological Sciences / Journal Canadien des Sciences Neurologiques.

[31] Gumprecht HK, Widenka DC, Lumenta CB (1999). BrainLab VectorVision neuronavigation system: technology and clinical experiences in 131 cases. Neurosurgery.

[32] Hasegawa H, Samuel M, Douiri A, Ashkan K (2014). Patients’ expectations in subthalamic nucleus deep brain stimulation surgery for Parkinson disease. World Neurosurg.

[33] Henry RG, Berman JI, Nagarajan SS, Mukherjee P, Berger MS (2004). Subcortical pathways serving cortical language sites: initial experience with diffusion tensor imaging fiber tracking combined with intraoperative language mapping. Neuroimage.

[34] Herbet G, Lafargue G, Moritz-Gasser S, Menjot de Champfleur N, Costi E, Bonnetblanc F, Duffau H (2015). A disconnection account of subjective empathy impairments in diffuse low-grade glioma patients. Neuropsychologia.

[35] Ille S, Ohlerth AK, Colle D, Colle H, Dragoy O, Goodden J, Robe P, Rofes A, Mandonnet E, Robert E, Satoer D, Viegas CP, Visch-Brink E, van Zandvoort M, Krieg SM (2021). Augmented reality for the virtual dissection of white matter pathways. Acta Neurochir (Wien).

[36] Incekara F, Smits M, Dirven C, Vincent A (2018). Clinical feasibility of a wearable mixed-reality device in neurosurgery. World Neurosurgery.

[37] Inoue D, Cho B, Mori M, Kikkawa Y, Amano T, Nakamizo A, Yoshimoto K, Mizoguchi M, Tomikawa M, Hong J, Hashizume M, Sasaki T (2013). Preliminary study on the clinical application of augmented reality neuronavigation. J Neurol Surg A Cent Eur Neurosurg.

[38] Ius T, Angelini E, Thiebaut de Schotten M, Mandonnet E, Duffau H (2011). Evidence for potentials and limitations of brain plasticity using an atlas of functional resectability of WHO grade II gliomas: towards a “minimal common brain”. Neuroimage.

[39] Jean WC (2022). Virtual and augmented reality in neurosurgery: the evolution of its application and study designs. World Neurosurgery.

[40] Jean WC, Britz GW, DiMeco F, Elmi-Terander A, McIntyre C (2021). Introduction. Virtual and augmented reality in neurosurgery: a timeline. Neurosurg Focus.

[41] Jean WC, Sack KD, Tsen AR (2022). Augmented-reality template guided transorbital approach for intradural tumors. Neurosurgical Focus: Video.

[42] Jung J, Lavrador JP, Patel S, Giamouriadis A, Lam J, Bhangoo R, Ashkan K, Vergani F (2019). First United Kingdom experience of navigated transcranial magnetic stimulation in preoperative mapping of brain tumors. World Neurosurg.

[43] Kalkofen D, Sandor C, White S, Schmalstieg D (2011) Visualization techniques for augmented reality. In. pp 65–98. 10.1007/978-1-4614-0064-6_3

[44] Kamada K, Todo T, Morita A, Masutani Y, Aoki S, Ino K, Kawai K, Kirino T (2005). Functional monitoring for visual pathway using real-time visual evoked potentials and optic-radiation tractography. Neurosurgery.

[45] Kantelhardt SR, Gutenberg A, Neulen A, Keric N, Renovanz M, Giese A (2015). Video-assisted navigation for adjustment of image-guidance accuracy to slight brain shift. Oper Neurosurg (Hagerstown).

[46] Katsevman GA, Greenleaf W, García-García R, Perea MV, Ladera V, Sherman JH, Rodríguez G (2021). Virtual reality during brain mapping for awake-patient brain tumor surgery: proposed tasks and domains to test. World Neurosurgery.

[47] Kersten-Oertel M, Gerard I, Drouin S, Mok K, Sirhan D, Sinclair DS, Collins DL (2015). Augmented reality in neurovascular surgery: feasibility and first uses in the operating room. Int J Comput Assist Radiol Surg.

[48] Khan OH, Mason W, Kongkham PN, Bernstein M, Zadeh G (2016). Neurosurgical management of adult diffuse low grade gliomas in Canada: a multi-center survey. J Neurooncol.

[49] Kockro RA, Tsai YT, Ng I, Hwang P, Zhu C, Agusanto K, Hong LX, Serra L (2009). Dex-ray: augmented reality neurosurgical navigation with a handheld video probe. Neurosurgery.

[50] Kosterhon M, Gutenberg A, Kantelhardt SR, Archavlis E, Giese A (2017). Navigation and image injection for control of bone removal and osteotomy planes in spine surgery. Oper Neurosurg (Hagerstown).

[51] Kuhnt D, Bauer MH, Nimsky C (2012). Brain shift compensation and neurosurgical image fusion using intraoperative MRI: current status and future challenges. Crit Rev Biomed Eng.

[52] Lavé A, Meling TR, Schaller K, Corniola MV (2020). Augmented reality in intracranial meningioma surgery: report of a case and systematic review. J Neurosurg Sci.

[53] Lavrador JP, Ghimire P, Gullan R, Ashkan K, Vergani F, Bhangoo R (2022). Preoperative and intraoperative anatomical-functional mapping in insular glioma surgery: integrated model to improve surgical outcome. J Neurosurg Sci.

[54] Lavrador JP, Gioti I, Hoppe S, Jung J, Patel S, Gullan R, Ashkan K, Bhangoo R, Vergani F (2020). Altered motor excitability in patients with diffuse gliomas involving motor eloquent areas: the impact of tumor grading. Neurosurgery.

[55] Lee C, Wong GKC (2019). Virtual reality and augmented reality in the management of intracranial tumors: a review. J Clin Neurosci.

[56] Lin HY, Hasegawa H, Mundil N, Samuel M, Ashkan K (2019). Patients’ expectations and satisfaction in subthalamic nucleus deep brain stimulation for Parkinson disease: 6-year follow-up. World Neurosurg.

[57] Liu T, Tai Y, Zhao C, Wei L, Zhang J, Pan J, Shi J (2020). Augmented reality in neurosurgical navigation: a survey. Int J Med Robot Comput Assist Surg.

[58] Luzzi S, Giotta Lucifero A, Martinelli A, Maestro MD, Savioli G, Simoncelli A, Lafe E, Preda L, Galzio R (2021). Supratentorial high-grade gliomas: maximal safe anatomical resection guided by augmented reality high-definition fiber tractography and fluorescein. Neurosurg Focus.

[59] Marigil M, Bernstein M (2018). Outpatient neurosurgery in neuro-oncology. Neurosurg Focus.

[60] Mascitelli JR, Schlachter L, Chartrain AG, Oemke H, Gilligan J, Costa AB, Shrivastava RK, Bederson JB (2018). Navigation-linked heads-up display in intracranial surgery: early experience. Oper Neurosurg (Hagerstown).

[61] Mazerand E, Le Renard M, Hue S, Lemée JM, Klinger E, Menei P (2017). Intraoperative subcortical electrical mapping of the optic tract in awake surgery using a virtual reality headset. World Neurosurg.

[62] Meola A, Cutolo F, Carbone M, Cagnazzo F, Ferrari M, Ferrari V (2017). Augmented reality in neurosurgery: a systematic review. Neurosurg Rev.

[63] Mikhail M, Mithani K, Ibrahim GM (2019). Presurgical and intraoperative augmented reality in neuro-oncologic surgery: clinical experiences and limitations. World Neurosurg.

[64] Milesi V, Cekic S, Péron J, Frühholz S, Cristinzio C, Seeck M, Grandjean D (2014). Multimodal emotion perception after anterior temporal lobectomy (ATL). Front Hum Neurosci.

[65] Mishra R, Narayanan MDK, Umana GE, Montemurro N, Chaurasia B, Deora H (2022) Virtual reality in neurosurgery: beyond neurosurgical planning. Int J Environ Res Public Health 19. 10.3390/ijerph19031719 10.3390/ijerph19031719 PMC8835688 35162742

[66] Mofatteh M (2021). Neurosurgery and artificial intelligence. AIMS Neurosci.

[67] Mofatteh M, Mashayekhi MS, Arfaie S, Chen Y, Hendi K, Kwan ATH, Honarvar F, Solgi A, Liao X, Ashkan K (2022). Stress, anxiety, and depression associated with awake craniotomy: a systematic review. Neurosurgery.

[68] Moher D, Liberati A, Tetzlaff J, Altman DG (2009). Preferred reporting items for systematic reviews and meta-analyses: the PRISMA statement. BMJ.

[69] Molina CA, Sciubba DM, Greenberg JK, Khan M, Witham T (2021). Clinical accuracy, technical precision, and workflow of the first in human use of an augmented-reality head-mounted display stereotactic navigation system for spine surgery. Oper Neurosurg (Hagerstown).

[70] Morshed RA, Young JS, Lee AT, Berger MS, Hervey-Jumper SL (2021). Clinical pearls and methods for intraoperative awake language mapping. Neurosurgery.

[71] Murcia D, D’Souza S, Abozeid M, Thompson JA, Djoyum TD, Ormond DR (2022). Investigation of asleep versus awake motor mapping in resective brain surgery. World Neurosurg.

[72] Nakajima R, Yordanova YN, Duffau H, Herbet G (2018). Neuropsychological evidence for the crucial role of the right arcuate fasciculus in the face-based mentalizing network: a disconnection analysis. Neuropsychologia.

[73] Park BS, Yoo MJ, Kim IH, Park JH, Park SH, Lee YJ, Park KM (2020). Alterations of gray matter volumes and connectivity in patients with tuberous sclerosis complex. J Clin Neurosci.

[74] Roberts DW, Strohbehn JW, Hatch JF, Murray W, Kettenberger H (1986). A frameless stereotaxic integration of computerized tomographic imaging and the operating microscope. J Neurosurg.

[75] Roethe AL, Rösler J, Misch M, Vajkoczy P, Picht T (2022). Augmented reality visualization in brain lesions: a prospective randomized controlled evaluation of its potential and current limitations in navigated microneurosurgery. Acta Neurochir (Wien).

[76] Rossi M, Puglisi G, Conti Nibali M, Viganò L, Sciortino T, Gay L, Leonetti A, Zito P, Riva M, Bello L (2022). Asleep or awake motor mapping for resection of perirolandic glioma in the nondominant hemisphere? Development and validation of a multimodal score to tailor the surgical strategy. J Neurosurg.

[77] Rowe AD, Bullock PR, Polkey CE, Morris RG (2001). “Theory of mind” impairments and their relationship to executive functioning following frontal lobe excisions. Brain.

[78] Ruis C (2018). Monitoring cognition during awake brain surgery in adults: a systematic review. J Clin Exp Neuropsychol.

[79] Rychen J, Goldberg J, Raabe A, Bervini D (2020). Augmented reality in superficial temporal artery to middle cerebral artery bypass surgery: technical note. Oper Neurosurg (Hagerstown).

[80] Sanai N, Mirzadeh Z, Berger MS (2008). Functional outcome after language mapping for glioma resection. N Engl J Med.

[81] Scherschinski L, McNeill IT, Schlachter L, Shuman WH, Oemke H, Yaeger KA, Bederson JB (2022). Augmented reality-assisted microsurgical resection of brain arteriovenous malformations: illustrative case. J Neurosurg Case Lessons.

[82] Scott H, Griffin C, Coggins W, Elberson B, Abdeldayem M, Virmani T, Larson-Prior LJ, Petersen E (2022) Virtual reality in the neurosciences: current practice and future directions. Front Surg 8. 10.3389/fsurg.2021.807195 10.3389/fsurg.2021.807195 PMC8894248 35252318

[83] Sharples S, Cobb S, Moody A, Wilson JR (2008). Virtual reality induced symptoms and effects (VRISE): comparison of head mounted display (HMD), desktop and projection display systems. Displays.

[84] Shinoura N, Suzuki Y, Yamada R, Tabei Y, Saito K, Yagi K (2010). Relationships between brain tumor and optic tract or calcarine fissure are involved in visual field deficits after surgery for brain tumor. Acta Neurochir.

[85] Suarez-Meade P, Marenco-Hillembrand L, Prevatt C, Murguia-Fuentes R, Mohamed A, Alsaeed T, Lehrer EJ, Brigham T, Ruiz-Garcia H, Sabsevitz D, Middlebrooks EH, Bechtle PS, Quinones-Hinojosa A, Chaichana KL (2020). Awake vs. asleep motor mapping for glioma resection: a systematic review and meta-analysis. Acta Neurochir (Wien).

[86] Sun GC, Wang F, Chen XL, Yu XG, Ma XD, Zhou DB, Zhu RY, Xu BN (2016). Impact of virtual and augmented reality based on intraoperative magnetic resonance imaging and functional neuronavigation in glioma surgery involving eloquent areas. World Neurosurg.

[87] Takami H, Khoshnood N, Bernstein M (2020). Preoperative factors associated with adverse events during awake craniotomy: analysis of 609 consecutive cases. J Neurosurg.

[88] Takami H, Venkatraghavan L, Bernstein M (2021). Perioperative factors affecting readmission after awake craniotomy: analysis of 609 consecutive cases. World Neurosurg.

[89] Toyooka T, Otani N, Wada K, Tomiyama A, Takeuchi S, Fujii K, Kumagai K, Fujii T, Mori K (2018). Head-up display may facilitate safe keyhole surgery for cerebral aneurysm clipping. J Neurosurg.

[90] Vadalà G, De Salvatore S, Ambrosio L, Russo F, Papalia R, Denaro V (2020). Robotic spine surgery and augmented reality systems: a state of the art. Neurospine.

[91] Weiss Lucas C, Pieczewski J, Kochs S, Nettekoven C, Grefkes C, Goldbrunner R, Jonas K (2021) The Cologne Picture Naming Test for Language Mapping and Monitoring (CoNaT): an open set of 100 black and white object drawings. Front Neurol 12. 10.3389/fneur.2021.633068 10.3389/fneur.2021.633068 PMC7966504 33746888

[92] Wolfson R, Soni N, Shah AH, Hosein K, Sastry A, Bregy A, Komotar RJ (2015). The role of awake craniotomy in reducing intraoperative visual field deficits during tumor surgery. Asian J Neurosurg.

[93] Wu B, Liu P, Xiong C, Li C, Zhang F, Shen S, Shao P, Yao P, Niu C, Xu R (2022). Stereotactic co-axial projection imaging for augmented reality neuronavigation: a proof-of-concept study. Quant Imaging Med Surg.

[94] Yee N, Bailenson JN, Urbanek M, Chang F, Merget D (2007). The unbearable likeness of being digital: the persistence of nonverbal social norms in online virtual environments. Cyberpsychol Behav.

[95] Young JS, Lee AT, Chang EF (2021). A review of cortical and subcortical stimulation mapping for language. Neurosurgery.

