# Supplementary material for: Augmented and virtual reality usage in awake craniotomy: a systematic review
Source: Neurosurg Rev. 2022 Dec 19;46(1):19. doi: 10.1007/s10143-022-01929-7 (PMC9760592; doi:10.1007/s10143-022-01929-7)
Supplement: Supplementary file 2 — Supplementary file2 (DOCX 15 KB) [file 10143_2022_1929_MOESM2_ESM.docx]

**Supplementary Table 1. Search Terms Used for Each Database**

| Data base and search results | Search term |
| --- | --- |
| PubMed  186 | (((((((augmented reality[Title/Abstract]) OR (virtual reality[Title/Abstract])) OR (extended reality[Title/Abstract])) OR (mixed reality[Title/Abstract])) OR (virtual simulation[Title/Abstract])) AND (awake craniotomy[Title/Abstract])) OR (awake brain surgery[Title/Abstract])) OR (awake neurosurgery[Title/Abstract])) OR (awake brain mapping[Title/Abstract])) OR (awake tumour resection[Title/Abstract]) |
| Scopus  27 | augmented AND reality OR virtual AND reality OR extended AND reality OR mixed AND reality OR virtual AND simulation AND awake AND craniotomy OR awake AND brain AND surgery OR awake AND neurosurgery OR awake AND brain AND mapping OR awake AND tumour AND resection |
| Web of Science  6 | ALL=((TI=((awake craniotomy OR awake brain surgery OR awake neurosurgery OR awake brain mapping OR awake tumour resection) AND (augmented reality OR virtual reality OR extended reality OR mixed reality OR virtual simulation))) OR AB=((awake craniotomy OR awake brain surgery OR awake neurosurgery OR awake brain mapping OR awake tumour resection) AND (augmented reality OR virtual reality OR extended reality OR mixed reality OR virtual simulation))) |
