# Supplementary material for: Augmented and virtual reality usage in awake craniotomy: a systematic review
Source: Neurosurg Rev. 2022 Dec 19;46(1):19. doi: 10.1007/s10143-022-01929-7 (PMC9760592; doi:10.1007/s10143-022-01929-7)
Supplement: Supplementary file 3 — Supplementary file3 (DOCX 17 KB) [file 10143_2022_1929_MOESM3_ESM.docx]

**Supplementary Table 2.** **A Summary of Studies Reviewed**

| **Study** | **Title** | **Journal** | **Country** | **Objectives** |
| --- | --- | --- | --- | --- |
| Mazerand et al., 2017 [45] | Intraoperative subcortical electrical mapping of the optic tract in awake surgery using a virtual reality headset | World Neurosurgery | France | To assess the visual field during awake craniotomy by developing the Functions’ Explorer based on a VR headset. |
| Bernard et al., 2018 [46] | Using a virtual reality social network during awake craniotomy to map social cognition: prospective trial | Journal of Medical Internet Research | France | To explain the experience of using a VR social network during awake craniotomy. |
| Delion et al., 2020 [47] | Immersing patients in a virtual reality environment for brain mapping during awake surgery: safety study | World Neurosurgery | France | To evaluate the tolerance, safety and experience of a VR headset in patients undergoing awake craniotomy. |
| Casanova et al., 2021 [33] | Immersive virtual reality and ocular tracking for brain mapping during awake surgery: prospective evaluation study | Journal of Medical Internet Research | France | To evaluate the feasibility and safety of a VR headset with an eye-tracking device capable of promoting an immersive visuospatial and social VR experience for patients undergoing awake craniotomy. |
| Ille et al., 2021 [48] | Augmented reality for the virtual dissection of white matter pathways | Acta Neurochirurgica | Germany | To evaluate a new method for fibre dissection using AR in AC a group which is experienced in cadaver white matter dissection courses and in vivo tractography. |
| Roethe et al., 2022 [49] | Augmented reality visualization in brain lesions: a prospective randomized controlled evaluation of its potential and current limitations in navigated microneurosurgery | Acta Neurochirurgica | Germany | To examine intraoperative visualisation parameters and clinical impact of AR in awake brain tumour surgery. |

AR, augmented reality; VR, virtual reality
